# Draft genome sequence of the BAL58 Betaproteobacteria representative strain LSUCC0117

**DOI:** 10.1128/MRA.00620-23

**Published:** 2023-10-13

**Authors:** Holly R. D. Stapelfeldt, V. Celeste Lanclos, Michael W. Henson, J. Cameron Thrash

**Affiliations:** 1 Department of Biological Sciences, University of Southern California, Los Angeles, California, USA; 2 Department of Geophysical Sciences, University of Chicago, Chicago, Illinois, USA; Montana State University, Bozeman, Montana, USA

**Keywords:** bacterioplankton, BAL58, high throughput culturing, LSUCC

## Abstract

Here, we present the draft genome sequence of strain LSUCC0117, a representative of the abundant aquatic BAL58 Betaproteobacteria group which we isolated from a coastal site in the northern Gulf of Mexico. The genome is estimated at over 99% complete, with a genome size of 2,687,225 bp.

## ANNOUNCEMENT

LSUCC0117 was isolated in artificial seawater media via dilution-to-extinction cultivation using an inoculum from Freshwater City (Louisiana, Gulf of Mexico, 29.53084, −92.32615) in March 2015 (1, 2). LSUCC0117 was closely related to several fellow isolates from the Louisiana State University Culture Collection (LSUCC), and close relatives outside the LSU culture collection included Betaproteobacterium BAL58 (99.56% identity, AY317112.1 [3]) and *Hydrogenophaga* sp. strain M7527 (98.74% identity, MT950111.1 [1, 2]). We chose to sequence LSUCC0117 due to its close identity to BAL58, which lies in a poorly studied yet abundant aquatic clade of obligately oligotrophic Betaproteobacteria found in freshwater-marine transition areas (2, 4), and to *Hydrogenophaga*, a genus of hydrogen-oxidizing bacteria with relevance to clean energy (5).

For sequencing, triplicate cryostocks of LSUCC0117 were revived in 50 mL of JW4 medium (2) in a polycarbonate flask and cultivated at room temperature until the late log phase. Cells were syringe filtered onto 0.2 µm polyethersulfone filters (Millipore Sigma, USA), and genomic DNA was extracted using a phenol-chloroform protocol with ethanol precipitation (https://dx.doi.org/10.17504/protocols.io.b5iiq4ce). Replicate DNA was combined, cleaned, and concentrated (Zymo Research, USC) and quantified with a Qubit fluorometer (Invitrogen, USA) using the HS dsDNA kit. Library preparation (KAPA HyperPlus library preparation kit, Kapa Biosystems, Inc., USA) and sequencing were completed at the University of Southern California Genome Core after size and quality analysis with the Agilent BioAnalyzer system. Paired-end 150 bp sequencing was performed with an Illumina NextSeq 550 using a midoutput flow cell, resulting in 5,757,661 read pairs.

Sequences were quality controlled using Trimmomatic v0.38 (6) with the following details: LEADING:20 TRAILING:20 SLIDINGWINDOW:13:20 MINLEN:40. Afterwards, we assembled reads using SPAdes v3.13.0 (7), followed by read mapping with the Burrows-Wheeler Aligner v0.7.17 (r1188) (8) and samtools v18.0.4 (9), and polishing with Pilon v. 1.22 (10) that produced the final assembly. Contigs less than 500 bp were manually removed. We evaluated the genome with Quast v5.2.0 (11) and CheckM2 v1.0.0 using “predict” (12), updated the taxonomy with GTDB-tk v2.1.1 using “classify_wf“ (13), and annotated the genome with the NCBI Prokaryotic Genome Annotation Pipeline (14). Default settings were used for all softwares unless otherwise noted.

**Fig 1:**
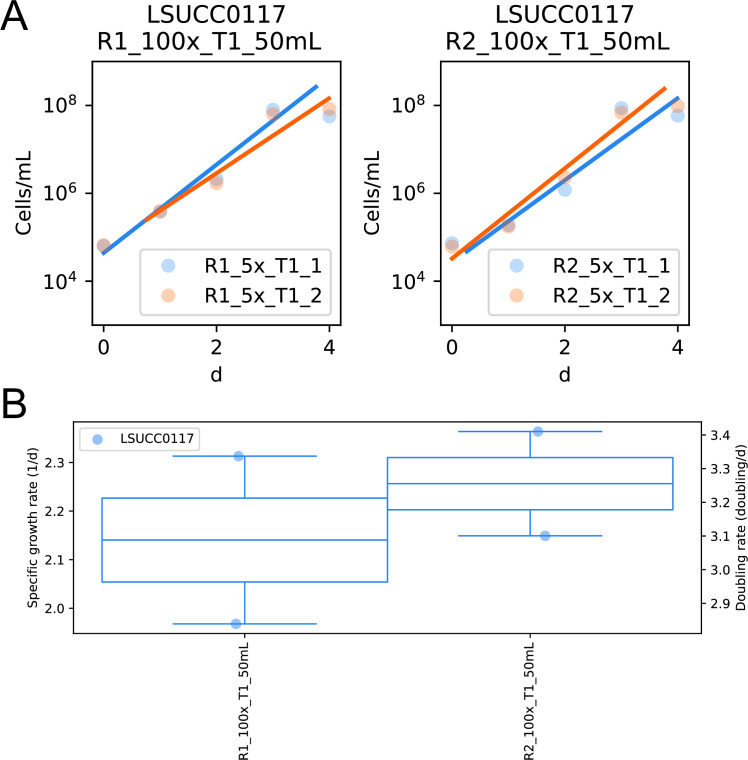
Growth curves (**A**) and rate calculations (**B**) for LSUCC0117. Cultures were grown in 5× JW4 medium at room temperature in 50 mL polycarbonate flasks, and cell density was measured with an Accuri C6 Plus flow cytometer (BD). Growth rates were calculated using sparse-growth-curve (15).

The LSUCC0117 draft genome is 2,687,225 bp in length with 13 scaffolds (N50: 433,083 bp), a 56.03% GC content, and a mean coverage of 545×. The genome encodes 2,624 putative genes, 2,553 of which were estimated to encode for proteins, with two copies each of the 5S, 16S, and 23S rRNA genes, and was predicted to be 99.99% complete, with 0.05% contamination. GTDB placed LSUCC0117 within the unclassified RS62 genus of the *Burkholderiaceae*. The mean growth rate of LSUCC0117 across two separate experiments was 3.17 ± 0.26 doublings per day at room temperature in 5× JW4 medium as determined by flow cytometry using an Accuri C6 Plus and growth calculations with sparse-growth-curve (15) (Fig. 1).

## Data Availability

This whole genome shotgun project has been deposited in DDBJ/ENA/GenBank under the accession no. JAUJFZ000000000. The version described in this paper is the first version, JAUJFZ010000000. The BioProject number is PRJNA988812, and the raw reads are available with SRA accession number SRX20918002. Cryostocks and/or live cultures of LSUCC0117 are available upon request.

## References

[1] Henson MW , Lanclos VC , Pitre DM , Weckhorst JL , Lucchesi AM , Cheng C , Temperton B , Thrash JC . 2020. Expanding the diversity of bacterioplankton isolates and modeling isolation efficacy with large-scale dilution-to-extinction cultivation. Appl Environ Microbiol 86:e00943-20. doi:10.1128/AEM.00943-20 32561583 PMC7440811

[2] Henson MW , Pitre DM , Weckhorst JL , Lanclos VC , Webber AT , Thrash JC . 2016. Artificial seawater media facilitate cultivating members of the microbial majority from the Gulf of Mexico. mSphere 1:e00028-16. doi:10.1128/mSphere.00028-16 PMC4894692 27303734

[3] Simu K , Hagström A . 2004. Oligotrophic bacterioplankton with a novel single-cell life strategy. Appl Environ Microbiol 70:2445–2451. doi:10.1128/AEM.70.4.2445-2451.2004 15066843 PMC383065

[4] Yeo SK , Huggett MJ , Eiler A , Rappé MS . 2013. Coastal bacterioplankton community dynamics in response to a natural disturbance. PLoS One 8:e56207. doi:10.1371/journal.pone.0056207 23409156 PMC3567041

[5] Kimura Z , Okabe S . 2013. Hydrogenophaga electricum sp. nov., isolated from anodic biofilms of an acetate-fed microbial fuel cell. J Gen Appl Microbiol 59:261–266. doi:10.2323/jgam.59.261 24005175

[6] Bolger AM , Lohse M , Usadel B . 2014. Trimmomatic: a flexible trimmer for Illumina sequence data. Bioinformatics 30:2114–2120. doi:10.1093/bioinformatics/btu170 24695404 PMC4103590

[7] Prjibelski A , Antipov D , Meleshko D , Lapidus A , Korobeynikov A . 2020. Using spades de novo assembler. Curr Protoc Bioinformatics 70:e102. doi:10.1002/cpbi.102 32559359

[8] Li H , Durbin R . 2009. Fast and accurate short read alignment with Burrows-Wheeler transform. Bioinformatics 25:1754–1760. doi:10.1093/bioinformatics/btp324 19451168 PMC2705234

[9] Li H , Handsaker B , Wysoker A , Fennell T , Ruan J , Homer N , Marth G , Abecasis G , Durbin R , 1000 Genome project data processing subgroup . 2009. Genome project data processing subgroup.The sequence alignment/map format and SAMtools. Bioinformatics 25:2078–2079. doi:10.1093/bioinformatics/btp352 19505943 PMC2723002

[10] Walker BJ , Abeel T , Shea T , Priest M , Abouelliel A , Sakthikumar S , Cuomo CA , Zeng Q , Wortman J , Young SK , Earl AM . 2014. Pilon: an integrated tool for comprehensive microbial variant detection and genome assembly improvement. PLoS One 9:e112963. doi:10.1371/journal.pone.0112963 25409509 PMC4237348

[11] Gurevich A , Saveliev V , Vyahhi N , Tesler G . 2013. QUAST: quality assessment tool for genome assemblies. Bioinformatics 29:1072–1075. doi:10.1093/bioinformatics/btt086 23422339 PMC3624806

[12] Chklovski A , Parks DH , Woodcroft BJ , Tyson GW . 2022. CheckM2: a rapid, scalable and accurate tool for assessing microbial genome quality using machine learning. bioRxiv. doi:10.1101/2022.07.11.499243 37500759

[13] Chaumeil P-A , Mussig AJ , Hugenholtz P , Parks DH . 2022. GTDB-TK V2: memory friendly classification with the genome taxonomy database. Bioinformatics 38:5315–5316. doi:10.1093/bioinformatics/btac672 36218463 PMC9710552

[14] Tatusova T , DiCuccio M , Badretdin A , Chetvernin V , Nawrocki EP , Zaslavsky L , Lomsadze A , Pruitt KD , Borodovsky M , Ostell J . 2016. NCBI prokaryotic genome annotation pipeline. Nucleic Acids Res 44:6614–6624. doi:10.1093/nar/gkw569 27342282 PMC5001611

[15] Cheng C , Thrash JC . 2021. Sparse-growth-curve: a computational pipeline for parsing cellular growth curves with low temporal resolution. Microbiol Resour Announc 10:e00296-21. doi:10.1128/MRA.00296-21 33986091 PMC8142577

